# Role of Tranexamic Acid in the Clinical Setting

**DOI:** 10.7759/cureus.8221

**Published:** 2020-05-21

**Authors:** Kyle Fischer, Elizabeth Awudi, Joseph Varon, Salim Surani

**Affiliations:** 1 Pharmacy, Texas A&M Rangel College of Pharmacy, Kingsville, USA; 2 Pharmacy, Corpus Christi Medical Center, Corpus Christi, USA; 3 Critical Care, United General Hospital, Houston, USA; 4 Critical Care, University of Texas Health Science Center, Houston, USA; 5 Internal Medicine, Corpus Christi Medical Center, Corpus Christi, USA; 6 Internal Medicine, University of North Texas, Dallas, USA

**Keywords:** tranexamic acid, antifibrinolytic agent, txa therapy update, crash-2, crash-3, trauma

## Abstract

Tranexamic acid (TXA) is labeled as an antifibrinolytic agent that decreases mortality, reduces blood loss after trauma or surgery, and lowers transfusion requirements in trauma patients with bleeding. This review of the literature is related to TXA use in a variety of settings, with a specific focus on trauma patients, to assess therapeutic efficacy and safety. As seen in large, randomized, placebo-controlled trials, TXA has been shown to decrease mortality over placebo in trauma patients, It is also noted to have good safety parameters upon administration and should be recommended for use in trauma patients with bleeding. Further studies are warranted for the use of TXA in gastrointestinal bleeding and pediatric trauma.

## Introduction and background

Tranexamic acid was initially described back in 1966 and since then, it has been used to control hemorrhage that stems from dental procedures, menstrual bleeding, and alter intra-operative hemodynamics [1]. Tranexamic Acid (TXA) is a prevalent anti-fibrinolytic drug that forms a reversible complex that displaces plasminogen from fibrin, which leads to fibrinolysis, thus leading to reduced surgical bleeding and perioperative blood transfusion [2]. TXA is a known inhibitor of lysine-binding sites on circulating plasmin, which, in turn, prevents the binding to fibrin and clot degradation. Due to its high affinity on the lysine site of plasminogen, TXA will displace plasminogen from the surface of fibrin. While plasmin may still be formed through conformational changes in plasminogen, the binding and dissolution of the fibrin matrix is inhibited [3]. Clinical data from Corticosteroid Randomisation after Significant Head Injury 2 (CRASH-2) and CRASH-3 trials exhibit significant potential toward reducing mortality among patients with hemorrhagic trauma [4-5].

Trauma has been known to be one of the leading causes of death in people younger than 40 years, with hemorrhage accounting for death in 30% of these patients [6]. TXA has been evaluated as a potential therapeutic option in indications like trauma, traumatic brain injury (TBI)/intracranial hemorrhage (ICH), pediatric trauma, gastrointestinal bleeding (GIB), postpartum hemorrhage (PPH), epistaxis, and disseminated intravascular coagulation (DIC) [7]. A review of the literature has been done to serve as a gauge when determining the safety and efficacy of TXA administration for certain indications, as concerns about the potential side effects of TXA remains among health care providers. Despite massive trials like the CRASH-2 and World Maternal Antifibrinolytic Trial (WOMAN) trials, there are still some questions needing answers [4,8].

Additionally, in this review, we analyzed the collections of ideas, literature, and guidelines with regard to the use of TXA in acute clinical settings where bleeding may lead to significant morbidity and mortality. This review will also accentuate trauma and other developing indications and assess the safety and efficacy of TXA.

## Review

Data source

**Figure 1:**
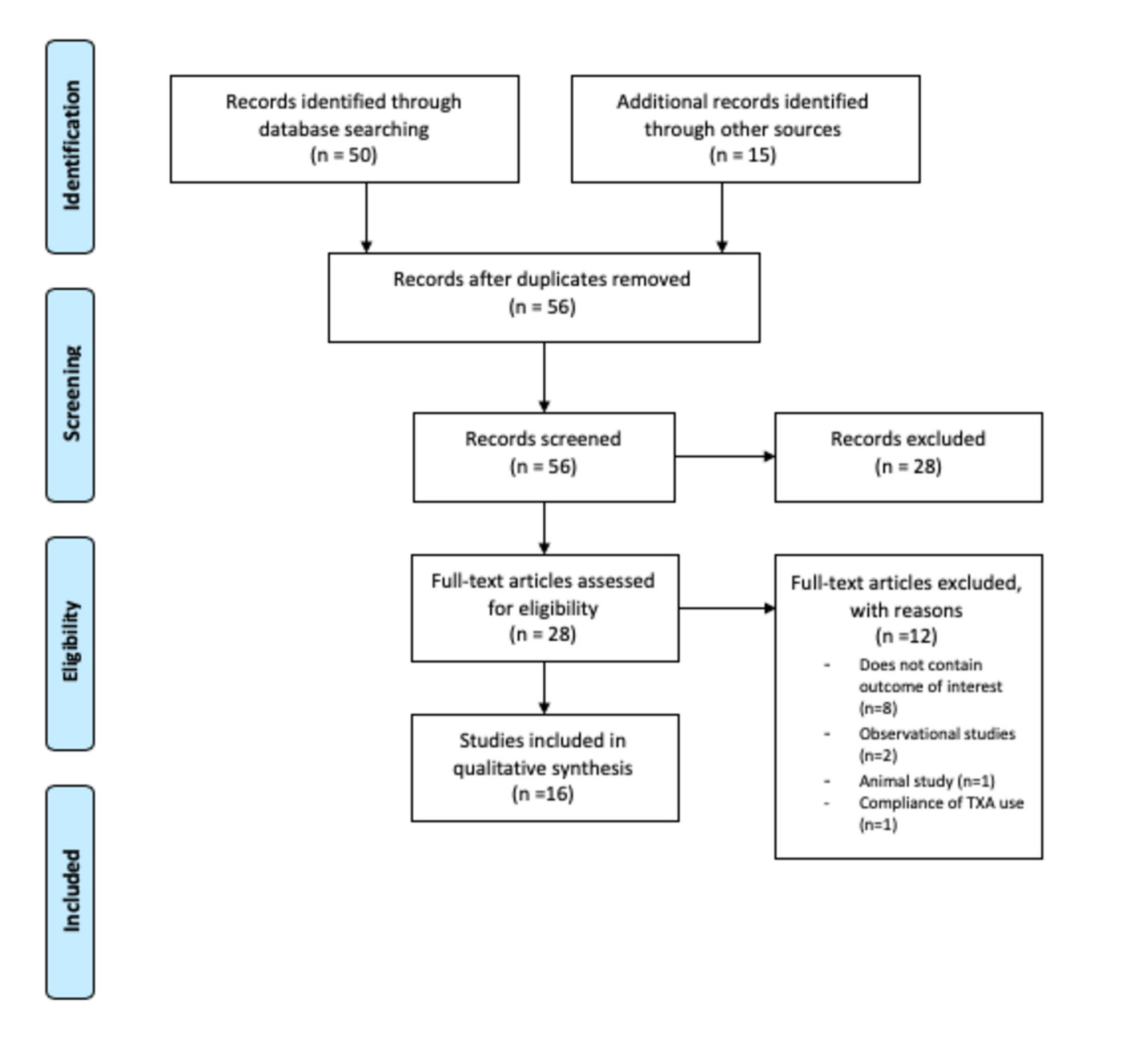
A Preferred Reporting Items for Systematic Reviews and Meta-Analyses (PRISMA) flowchart of the literature and search strategy of studies

We performed a systematic literature search on topics that compared the safety and efficacy of TXA administration in a variety of potential indications. We searched the PubMed and Google Scholar databases with the keywords “tranexamic acid,” “CRASH-2 Trial,” “CRASH-3 Trial,” “WOMAN Trial,” “HALT-IT Trial,” “TXA and trauma patients,” “TXA safety and efficacy,” and “TXA and bleeding”. We also performed hand-sampling of references from the included studies. The last search was run on January 3, 2020. A Preferred Reporting Items for Systematic Reviews and Meta-Analyses (PRISMA) flowchart of the literature and search strategy of the studies is shown in Figure 1.

Eligibility criteria

The inclusion criteria for this study are studies comparing the safety and efficacy of TXA administration in patients with TBI/ICH, pediatric trauma, GIB, PPH, epistaxis, and DIC. All related clinical research/original articles and excluded animal studies, case reports, and review articles that have reported the safety and efficacy outcomes were included. No language, publication date, or publication status restrictions were imposed. Participants of any age receiving TXA were considered and trials comparing the beneficial and harmful effects of TXA administration were studied. In addition, we hand-searched content pages like the World Health Organization (WHO), UK Defense Medical Service’s transfusion protocol, and the UK Royal College of Pediatrics evidence statement on TXA administration. Eligibility assessment was performed by two researchers (KF and EA) in an unblinded, standardized manner. Any discrepancies were resolved via discussion.

Data extraction

Data extraction was performed by three authors (KF, EA, and SS) using a standardized extraction form that includes author, year of publication, study design, number of cases/controls, findings, and positives/negatives. The primary outcome measures were mortality benefit and adverse effect profile.

Results of included studies

TXA has been used for upper GIBs and not lower GIBs and a recent systematic review of seven clinical trials of TXA for use in upper GIBs showed a significant reduction in mortality, but the adverse events were not reported [9-10]. Table 1 summarizes the seven clinical trials and their findings. Efficacy and safety data is insufficient at this time to recommend the use of TXA in GIBs [11-12]. However, a large multi-center study that is currently underway by the name of Tranexamic Acid for Acute Gastrointestinal Bleeding (HALT-IT) should provide clarification and sufficient data for this indication [13].

**Table 1:** Tranexamic acid for gastrointestinal bleed RCT: randomized control trials; TXA: tranexamic acid

Authors	Study Design	Number of Cases/Controls	Positive/Negative Findings
Cormack et al., 1973 [14]	Double-blind RCT	150 patients (76 TXA and 74 placebos)	No statistical differences were found in continued bleeding, recurrence of bleeding, and need for further transfusion or surgery
Biggs et al., 1976 [15]	Double-blind RCT	200 patients (103 TXA and 97 placebos)	-No statistically significant difference in mortality
Engquist et al., 1979 [16]	Double-blind RCT	149 patients (76 TXA and 73 placebos)	-No ADR reported -TXA group needed less blood and fewer operations compared to the control group -Statistical significance was not reached except with respect to blood transfused on the second day of treatment
Bergqvist et al., 1980 [17]	Double-blind RCT	43 patients (21 TXA and 22 placebos)	-No statistical significance was found due to the small sample size
Barer et al., 1983 [18]	Double-blind RCT	775 patients (256 TXA, 259 cimetidine, and 260 placebos)	-Mortality was lowest with the TXA group p=0.0092 -Authors can’t conclude why low mortality was shown in the TXA group
Holstein et al., 1987 [19]	Double-blind RCT	154 patients (72 TXA and 82 placebos)	-Blood transfusion requirements were reduced in TXA group p=0.018 -Fewer patients re-bleed in the TXA group p=0.097 -Three patients in the TXA group had series complications compared to none in the placebo
Hawkey et al., 2001 [20]	Double-blind RCT	228 patients (47 TXA, 58 lansoprazole, 55 placebos, 58 both drugs)	-Similar number of patients suffered re-bleeding in all treatment groups -No significant influence on the risk of re-bleeding -No statistical significance was reached on any of the trial treatments on clinical endpoints

Epistaxis has been considered as an indication for the administration of TXA. In 2013, a randomized controlled trial of 216 patients looked at the application of TXA in idiopathic anterior epistaxis. This trial showed a substantial benefit of TXA for bleeding, discharge time, and re-bleeding rates. Bleeding was noted to cease within 10 minutes in 71% of the TXA group versus 31.2% in the anterior nasal packing group (OR 2.28). Discharge within two hours occurred in 95.3% of the TXA group versus just 6.4% in the anterior nasal packing group. Re-bleeding rates were 4.7% versus 11% for the TXA and anterior packing group in the first 24 hours, respectively [21]. Another study revealed that there is insufficient evidence to support the use of topical intranasal TXA for spontaneous epistaxis in hemodynamically stable patients in the emergency department (ED) [22].

DIC has been another indication of where TXA may have an effect. However, it has been noted that by blocking the fibrinolytic system, as TXA does, there may be an increase in thrombotic complications, thus antifibrinolytics are routinely not used in the management and treatment of DIC. Before completely counting TXA out for this indication, one guideline has given consideration to TXA in patients who present with severe bleeding associated with a hyper fibrinolytic state [23].

TXA has also been floated around to reduce uterine blood loss in women with menorrhagia, but a randomized control trial showed the efficacy of TXA for intractable postpartum hemorrhage (PPH) to be marginal. In 2013, a case-control study suggested that TXA notably reduced blood loss for up to two hours peripartum in primiparous C-sections (TXA group average 42.7 mL blood loss versus placebo control average 80.2 mL) [24]. Although the study was not adequately powered to address safety issues, the adverse effects were noted to be mild. The guidelines from the World Health Organization (WHO) suggested that TXA may be used as an alternative if others fail. Evidence is of low quality. In the UK, TXA is considered for the treatment of intractable PPH [24]. Further studies are warranted to determine TXA safety and efficacy for this indication, and the WOMAN trial was designed to answer such questions [8]. In the WOMAN trial, the use of TXA in postpartum hemorrhage revealed no difference in the primary outcome, death within 42 days, or hysterectomy (TXA 5.3% versus placebo 5.5%) [7]. They was also a statistically significant difference in death due to postpartum hemorrhage: 1.5% in the TXA group versus 1.9% in the placebo group (p=0.045) [8]. This trial showed through a similar protocol to CRASH-2 that TXA was indeed effective in postpartum hemorrhage, but the administration of TXA beyond three hours of trauma or delivery was associated with an increase in mortality compared to placebo. Nevertheless, the WOMAN trial did not present a subgroup analysis examining the patients with major bleeding, thus the efficacy of TXA in the subset of patients with serious bleeding was left with some concern upon completion of the trial. Subsequent studies were examined to clarify these concerns with the CRASH-3 trial as well as compare the WOMAN trial to TXA landmark studies [5]. Table 2 shows the mortality results of the CRASH-2, CRASH-2 followup, CRASH-3, and WOMAN trials [25]. Table 3 also presents some landmark trials as they pertain to TXA.

**Table 2:** Mortality results of CRASH-2, CRASH-2 followup, CRASH-3, and WOMAN CI: confidence interval; CRASH: Clinical Randomization of an Antifibrinolytic in Significant Hemorrhage; GCS: Glasgow Coma Scale/Score; pt.: patients; RR: relative risk; TXA: tranexamic acid; WOMAN: World Maternal Antifibrinolytic

Studies	Main Findings
CRASH-2 [4]	**Any cause of death (28 days): TXA 1463 (14.5%) vs placebo 1613 (16%); RR = 0.91 (95% CI, 0.85-0.97); P = 0.0035 **Bleeding: TXA 489 (4.9%) vs 574 (5.7%); RR = 0.85 (95% CI, 0.76-0.96); P = 0.0077 **Patient in shock (BP systolic≤75 mm Hg): TXA 478 of 1562 patients. (30.6%) vs 562 of 1599 pt. (35.1%): RR = 0.87; 95% CI (0.76-0.99)
CRASH-2 Follow-Up [26]	**TXA within 1 h after trauma: TXA 198 of 3747 pt. (5.3%) versus placebo 286 of 3704 pt. (7.7%); RR = 0.68 (95% CI, 0.57–0.82); P < .0001. **TXA 1–3 h after trauma: TXA 147 of 3037 pt. (4.8%) versus placebo 184 of 2996 pt. (6.1%); RR = 0.79 (95% CI, 0.64–0.97a); P = .03
CRASH-3 [5]	**Risk of head injury-related death within 3 hours from injury: TXA (18.5%) vs placebo (19.8%); RR = 0.94 (95% CI, 0.86-1.02) **Pre specified sensitivity analysis removing patients with a GCS of 3 and bilateral un reactive pupils at baseline: TXA (12.5%) vs placebo (14%); RR 0.89 (95% CI, 0.80-1.00) **Risk of head injury-related death in patients with mild-to-moderate head injury (GCS 9-15): TXA 166 of 2846 (5.8%) vs placebo 207/2769 (7.5%); RR 0.78 (95% CI, 0.64-0.95) **Risk of head injury-related death in patients with severe head injury (GCS 3-8): TXA 689 of 1739 (39.6%) vs placebo 685 of 1710 (40.2%); RR 0.99 (95% CI, 0.91-1.07)
WOMAN [8]	**Death due to bleeding: TXA 155 (1.5%) versus placebo 191 (1.9%); RR = 0.81 (95% CI, 0.65–1.0a); P = .045 **Death if TXA within 3 h: TXA 89 (1.2%) versus placebo 127 (1.7%); RR = 0.69 (95% CI, 0.52–0.91a); P = .008 **Laparotomy to control bleeding: TXA 82 (0.8%) versus placebo 127 (1.3%); RR = 0.64 (95% CI, 0.49–0.85); P = .002

**Table 3:** Tranexamic acid landmark studies CRASH: Clinical Randomization of an Antifibrinolytic in Significant Hemorrhage; TXA: tranexamic acid

YEAR	TITLE	MAIN RESULTS
2010	CRASH-2 [4]	TXA recommended for all trauma patients
2011	CRASH-2 Follow-Up [26]	Time dependency of TXA effect
2012	MATTERs [27]	TXA accepted in the WHO list of “Essential Medicines”
2014	Israel Defense Forces [28]	Likely benefit in the civilian sector
2015	Cole et al. [29]	TXA effective only for “severely injured shocked patients”. Limitation of TXA indication on patients with “severe hemorrhage”
2016	WOMAN [8]	TXA effective in postpartum hemorrhage
2017	Gayet-Ageron et al. [30]	Survival benefit with TXA decreased by 10% for every 15 min of treatment delay until 3 h
2018	Cal-PAT [31]	Prehospital application of TXA is effective
2019	CRASH-3 [5]	TXA administration to patients with TBI within 3 h of injury reduces head injury-related death, with no evidence of adverse effects or complications

One indication that is becoming more popular among healthcare providers is if the TXA is effective and safe for use in pediatric trauma. Currently, there are no studies on pediatric trauma, but due to the success of TXA exhibited in the adult population and the successful use in pediatric non-trauma surgical settings, there have been some signals suggesting that the development of protocols should be implemented in adolescents sooner rather than later [32-33]. A better safety profile can be linked to this population, assuming the thromboembolic complications to be lower in the children, as their baseline cardiovascular risk is lower [23].

Recently, there has been a lot of association with TXA in trauma patients since the CRASH-2 trial, which was a large, randomized, placebo-controlled study that investigated the use of TXA in adult trauma patients at risk of hemorrhage [4]. The investigators saw that a loading dose of 1 g intravenous (IV) plus another 1 g IV over the next eight hours significantly reduced the all-cause mortality and mortality from bleeding [4]. The results of the CRASH-2 trial are highly generalizable and a benefit from TXA was found despite the reduction of the effect by the large number of patients that did not receive a blood transfusion or surgery. One subgroup that was isolated from the CRASH-2 trial were patients with isolated head trauma. Within the trial, it was noted that 270 patients enrolled had multi-trauma, including both extracranial bleeding and traumatic brain injury (TBI). The Intracranial Bleeding Study was a randomized, double-blind, placebo-controlled trial within the CRASH-2 trial that reviewed the effect of TXA on this subset of patients and the results were not statistically significant, but all clinical trends within the pragmatic study favored TXA over placebo [34].

With that being said, the largest randomized controlled trial for TXA use in isolated TBI has been the CRASH-3 trial [5]. The CRASH-3 trial planned to assess TXA use in isolated TBI and, more specifically, its effects on mortality, disability, and safety. The CRASH-3 trial was designed to further investigate the appropriateness of TXA for patients with traumatic brain injury, as it was a large, pragmatic, double-blind randomized controlled trial involving 175 hospitals in 29 countries, with a target enrollment of 10,000 patients. The authors enrolled adult patients with TBI, within three hours of injury, with a Glasgow Coma Scale (GCS) score of less than or equal to 12, or any intracranial bleeding on computed tomography (CT) scan that did not show signs of major extracranial hemorrhage. The patients in this trial received the same protocol for TXA administration as in the CRASH-2 trial [4-5]. During this trial, the protocol was corrected slightly to a time window of recruitment from less than eight hours post-injury to less than three hours. This was done based on data showing no benefit and potential harm in TXA administration greater than three hours after a trauma incident [5]. Paralleling the WOMAN trial, it was reported that there was no difference in the primary outcome, rate of head injury-related death in hospital within 28 days of injury, 18.5% in the TXA group versus 19.8% in the placebo group (RR 0.94 [5]. The authors also studied the subset of patients with neurological injury (patients with a GCS score of three, or bilateral nonreactive pupils at baseline). They were found to have a statistically significant 1.5% absolute reduction in head injury-related death (12.5% in the TXA group versus 14% in the placebo group) [5,8]. The main findings are depicted in Table 2 and one can compare the similarities to the other landmark trials involving TXA.

The trend across multiple studies are pointing in a positive direction and the data from the CRASH-3 trial were similar to the results obtained from the CRASH-2 trial of TXA in patients with traumatic extracranial bleeding. The insignificant number of adverse events is also positive, thus helping TXA find a place in ICH therapy. At this stage, it is difficult to make a complete recommendation for effectiveness, but it would also be incorrect to say TXA is ineffective. Thus, additional clinical trials are needed to determine the true role that TXA plays.

Discussion

To summarize the current literature, this review of TXA identified and analyzed prominent trials and literature to define TXA’s safety and efficacy in various indications. Indications like GIB, epistaxis, DIC, postpartum hemorrhage, pediatric trauma, adult trauma at risk of hemorrhage, and TBI were all explored. Through the review of literature conducted, it was found that TXA had a potential benefit in postpartum hemorrhage and is now well-established in the management of bleeding trauma patients [8]. CRASH-2, CRASH-3, and ensuing trials have shown that TXA is effective and that the benefits are paramount when given as soon as possible [4-5]. There is still a lack of benefit or an unclear role for TXA use in indications for GIB, epistaxis, DIC, and pediatric trauma populations.

Given this emerging body of evidence, we have seen WHO guidelines in the UK suggest that TXA is a potential alternative to initiate for intractable PPH as well as included in the WHO’s list of essential medicines [35]. TXA has also been found to be included in various trauma protocols worldwide, such as the UK Defense Medical Service’s massive transfusion protocol, the UK Royal College of Pediatrics, and Child Health’s. 

In patients with GIB, we are awaiting data from the HALT-IT study, but after the publication of CRASH-3, there were some indicators showing a reduction in GIB among patients treated with TXA (relative risk 0.68, confidence interval 0.4-1.14). This data may foretell the results of the HALT-IT trial, which is examining the use of TXA in GIB. In regard to the use of TXA in epistaxis, a review of 33 papers in 2014 showed that there is still insufficient evidence to support the use of topical intranasal TXA for this indication. In patients that present with DIC, the British Committee for Standards in Hematology has given consideration to TXA for patients with severe bleeding associated with hyper-fibrinolytic states, however, more studies are needed to assess the true efficacy and safety of TXA in this patient population. Healthcare providers should use extreme caution or avoid the use of TXA in DIC patients altogether. In patients that have uterine blood loss due to menorrhagia, TXA has shown to be effective and safe, but the administration of TXA after three hours of trauma or delivery was associated with an increase in mortality in the WOMAN trial [8]. Data from this large randomized controlled trial can be used by healthcare providers to use TXA in treating postpartum hemorrhage within three hours and to avoid using it after three hours of trauma or delivery. Studies have not looked at TXA use in pediatric trauma but since the benefits look so promising and due to recent success in the adult population, there may be some benefit in extrapolating the resultant effect in adults to the pediatric trauma population, as it would be very hard to get a trial with enough pediatric patients to provide a statistically significant result. So, it may be beneficial to use it in pediatric patients with TBI just like adults, although there is not sufficient data to support this claim and in the authors' opinion, it would be wise to use the best clinical judgment when dealing with pediatric patients who present with TBI.

In the CRASH-3 trial that examined the use of TXA in TBI, the primary endpoint fell marginally short of reaching statistical significance, but it could possibly be linked to the inclusion of patients who were severely injured and unlikely to survive regardless of any therapy. The subgroup analysis showed benefit from TXA among patients with a moderate head injury, though further study is suggested.

There has been some momentum building up for TXA use, specifically in bleeding adult trauma patients and adult patients with TBI. As evidence from the latest trial CRASH-3, the sooner TXA is given, the more favorable outcomes are expected. In concordance with CRASH-3, it can be said that the administration of TXA would have no impact on non-head injured deaths. So we are left with the task to try and clarify if we should give TXA to every trauma patient or who exactly is going to reap the benefits of TXA. From our review, we found that TXA works in bleeding related to trauma and in isolated head injuries. However, as mentioned above, there is not sufficient data on efficacy in multi-system trauma or children. It may be reasonable to extrapolate the safety in polytrauma patients and although there is some consensus to administer TXA to pediatric trauma patients at a reduced dose, there is unlikely to be a separate trial for either group in the foreseeable future.

Furthermore, TXA use is a promising and affordable option for numerous indications, especially in adults with trauma and TBI; however, the quality of evidence supporting all indications is not without limitations. The consistency of treatment outcomes for TXA in adult trauma hemorrhage and TBI has shown to benefit in patients with a less severe injury and showing a strong safety profile. Data stemming from multiple, large, pragmatic trials can further justify TXA use. Possible adverse events were thoroughly evaluated and TXA was found not to cause further harm. However, there may be a type I error due to the size of the subgroup in which statistical benefit was shown in regards to the CRASH-3 trial and future randomized controlled trials comparing TXA to the standards of care are merited in patients with GIB and pediatric trauma in order to provide further evidence of treatment outcomes. The HALT-IT trial looked at these questions, but unfortunately, due to the challenges in enrolling pediatric trauma patients to measure TXA efficacy, we may not have a feasible answer in the foreseeable future.

## Conclusions

TXA is a safe and economically viable option that displays a significant mortality benefit. Through multiple studies, there have been no apparent serious safety issues, and the early administration of TXA is coupled with the most benefit. TXA should not be given three hours post-injury due to the increase in mortality that has been noted. In the numerous trauma practice environments, TXA emerges as having equal benefit, and healthcare providers should use their best clinical judgment when administering it. Upon completion of the HALT-IT trial, we expect to see TXA’s true efficacy in GIB. Further randomized studies are also suggested to expand its role in other indications.
